# Clinical Meaning of Stromal Tumor Infiltrating Lymphocytes (sTIL) in Early Luminal B Breast Cancer

**DOI:** 10.3390/cancers15102846

**Published:** 2023-05-20

**Authors:** Esmeralda García-Torralba, Miguel Pérez Ramos, Alejandra Ivars Rubio, Esther Navarro-Manzano, Noel Blaya Boluda, Pilar de la Morena Barrio, Elisa García-Garre, Francisco Martínez Díaz, Asunción Chaves-Benito, Elena García-Martínez, Francisco Ayala de la Peña

**Affiliations:** 1 Department of Medical Oncology, Hospital Universitario Morales Meseguer, 30008 Murcia, Spain; esmeralda.garciat@um.es (E.G.-T.); mariaalejandra.ivars@um.es (A.I.R.); noel.blaya@um.es (N.B.B.); pdb89e@ad.sms.carm.es (P.d.l.M.B.); elisa.garcia2@carm.es (E.G.-G.); helenagarciam@gmail.com (E.G.-M.); 2 Department of Medicine, Medical School, University of Murcia, 30001 Murcia, Spain; esther.navarro3@um.es; 3 Instituto Murciano de Investigación Biosanitaria, IMIB, 30120 Murcia, Spain; 4 Department of Pathology, Hospital Universitario Morales Meseguer, 30008 Murcia, Spain; miguel.perez3@carm.es (M.P.R.); mariaa.chaves@carm.es (A.C.-B.); 5 Centro Regional de Hemodonación, 30003 Murcia, Spain; 6 Department of Pathology, Hospital Universitario Reina Sofía, 30003 Murcia, Spain; fmdiaz@um.es; 7 Department of Pathology, Medical School, University of Murcia, 30001 Murcia, Spain; 8 Medical School, Universidad Católica San Antonio, 30107 Murcia, Spain

**Keywords:** breast cancer, lymphocyte, TIL, prognostic factor, predictive factor, neoadjuvant chemotherapy, adjuvant chemotherapy, survival, pathologic complete response

## Abstract

**Simple Summary:**

Stromal tumor infiltrating lymphocytes (sTIL) are a validated predictive and prognostic biomarker in non-luminal breast cancer. Our aim was to evaluate their clinical relevance in luminal (hormone receptor positive, HER2 negative) early breast cancer. Our results show that, although sTIL are associated with a better response to neoadjuvant chemotherapy, they are also associated with worse biological features (proliferation, higher stage) and poorer prognosis in luminal B breast cancer. TIL might improve prognostic stratification and contribute to therapeutic decision-making in the early high-risk setting of luminal B breast cancer.

**Abstract:**

Luminal breast cancer (BC) is associated with less immune activation, and the significance of stromal lymphocytic infiltration (sTIL) is more uncertain than in other BC subtypes. The aim of this study was to investigate the predictive and prognostic value of sTIL in early luminal BC. The study was performed with an observational design in a prospective cohort of 345 patients with predominantly high-risk luminal (hormone receptor positive, HER2 negative) BC and with luminal B features (*n* = 286), in which the presence of sTIL was analyzed with validated methods. Median sTIL infiltration was 5% (Q1–Q3 range (IQR), 0–10). We found that sTIL were associated with characteristics of higher biological and clinical aggressiveness (tumor and lymph node proliferation and stage, among others) and that the percentage of sTIL was predictive of pathologic complete response in patients treated with neoadjuvant chemotherapy (OR: 1.05, 95%CI 1.02–1.09, *p* < 0.001). The inclusion of sTIL (any level of lymphocytic infiltration: sTIL > 0%) in Cox regression multivariable prognostic models was associated with a shorter relapse-free interval (HR: 4.85, 95%CI 1.33–17.65, *p =* 0.016) and significantly improved its performance. The prognostic impact of sTIL was independent of other clinical and pathological variables and was mainly driven by its relevance in luminal B BC.

## 1. Introduction

Breast cancer (BC) is the most common malignancy and the leading cause of cancer death in women [1]. The most frequent subtype of BC, accounting for 70% of cases, is luminal BC, which is dependent on estrogenic transcriptional programs and is usually identified in the clinical setting as the subgroup of hormone receptor (HR)-positive tumors with an absence of HER2 amplification (HER2 negative) [2]. The recent introduction of immunotherapy as a treatment strategy in BC has so far involved only triple-negative (HR negative, HER2 negative) tumors, characterized by increased immune activation and by a higher presence of tumor infiltrating lymphocytes in the tumor stroma (sTIL) [3]. In contrast, in luminal BC, immune-activated subtypes are less frequent [4], lymphocyte infiltration is lower [5] and previous efforts to introduce therapeutic strategies aimed at enhancing the immune response against tumors have shown poor results in advanced disease [6,7,8]. More recent studies with a combination of chemotherapy and immunotherapy in early disease have yielded somewhat better results in luminal tumors [9], especially in those with high proliferation and endocrine resistance. 

The prognostic significance of lymphocyte infiltration in HR+ HER2- BC is also less clear than in HR-negative and HER2-positive tumors, in which higher percentages of sTIL are associated with better disease-free survival (DFS) and overall survival (OS) [10]. This association is not consistently observed in luminal tumors, in which although higher lymphocyte infiltrates are associated with higher genomic risk (recurrence score) [11], no differences in survival are observed [12]. The prognostic impact of sTIL in luminal BC has indeed been inconsistent between publications, with some studies showing no difference in DFS or OS [10,12,13] and other studies finding a negative impact on OS with no difference in DFS [5]. In patients treated with neoadjuvant chemotherapy, a recent meta-analysis, prior to the publication of the largest series [13], concluded that elevated sTIL was associated with shorter OS in tumors of the luminal subtype [14], although no differences in DFS by sTIL level were found either. 

The different prognostic effect of sTIL in luminal BC is also supported by other series analyzing specific lymphocyte populations such as CD8+ TILs. Although these studies have shown varying results [15,16], higher CD8+ TIL infiltration does not seem to predict better outcomes in HR+ HER2- tumors. This observation strongly contrasts with the findings in other subtypes, where greater lymphocyte infiltration is associated with an enhanced antitumor immune response and a more favorable outcome [12]. Mechanistic explanations for the different meanings of sTIL in luminal BC are lacking: a differential effect of anthracycline-based chemotherapy was proposed for this group, with an increased benefit of treatment in HR+ HER2- tumors without CD8+ infiltration [15], but statistical significance was not reached. No further analyses have supported this concept, with some data even showing reduced DFS after chemotherapy in patients with intermediate TIL infiltration [13]. Association of sTIL with higher proliferation, lower differentiation and higher genomic grade [13], which are well-known prognostic factors in luminal BC, are also potential explanations, and some data from multivariate models might support this concept [15]. The association of lymphocyte infiltration with lower HR expression has been suggested by prior reports, but could not be demonstrated in other cohorts of luminal BC patients [16]. Finally, other biological features, such as *PIK3CA* mutated status and differences in FOXP3+ populations in the tumor microenvironment, have also been proposed as potential explanations of the diverse prognostic impact of lymphocyte infiltration in luminal BC [16].

The above cited factors might account at least for some of the differences of prior works, which could also be driven by differences in the treatment administered (especially neoadjuvant or adjuvant chemotherapy), in the statistical adjustment for other prognostic factors or even in the method of sTIL assessment. Taken together, there remains a significant uncertainty about the clinical meaning of lymphocytic infiltration in HR+/HER2-negative BC. 

This lack of impact of sTIL on DFS, together with the uncertainty about its influence on OS and pathologic complete response (pCR), and with the general difficulties presented by sTIL in its use as a clinical biomarker [17], have limited its applicability in early luminal BC. However, the results relating lymphocyte infiltration to higher proliferation [18] and lower HR expression [19], and those relating them to the luminal B BC subtype [20], could create differences in the clinical significance and utility of sTIL in luminal A and B tumors. Previous work has also pointed out different effects of sTIL on DFS in luminal BC depending on the level of Ki67, with negative prognostic effects in the low proliferation group [21] and better distant DFS in high-proliferating tumors, especially when the latter are treated with chemotherapy [18]. This interaction between chemotherapy (predominantly used in luminal B disease), proliferation and sTIL might also be relevant for understanding the role of sTIL and its potential variability between luminal A and B tumors. Thus, the considerable heterogeneity in sTIL distribution in luminal tumors might be translated to differences in its clinical significance.

Considering the potential relevance of sTIL to define immunotherapy-based approaches in patients with high-risk luminal BC and that further insights into the clinical correlations of immune microenvironment in luminal disease are needed to facilitate prognostic stratification in these patients, the aim of this work was to analyze the clinical significance of sTIL, in terms of relapse-free and overall survival, in luminal BC, taking into account the surrogate immunohistochemical definition of luminal A and B subtypes. 

## 2. Materials and Methods

### 2.1. Study Design

An observational single-center cohort of 345 women with early luminal (defined as HR positive, HER2 negative) BC (2012–2020) was analyzed. This cohort was obtained from a previous cohort of 1006 consecutive breast cancer cases, including 651 cases of luminal BC (Supplementary Figure S1) included in a translational study in which most patients received chemotherapy. Inclusion criteria for this study were positive expression of hormone receptors (HR), non-amplified HER2 (HER2-), availability of pre-treatment core biopsy for sTIL evaluation and signed informed consent for the study. Treatment was performed according to standard clinical practice [22]. The study was approved by the IRB (CEIC Hospital Morales Meseguer; code EST08/21).

### 2.2. Pathologic Evaluation and sTIL Analysis

Pre-treatment stromal tumor infiltrating lymphocytes (sTIL) were measured by an expert breast pathologist (MPR) blinded to the patients’ outcomes. Published standard methods from the International sTIL Working Group were followed for sTIL evaluation [23,24]. sTIL was expressed as the percentage of TIL in the intratumoral stromal area of H&E stained slides from a diagnostic core breast biopsy. Briefly, the whole stromal compartment within the borders of the invasive tumor was considered for sTIL quantification, and the percentage of area occupied by lymphocytes (related to the total area of stromal tissue, not to the number of cells) was estimated as a continuous variable. Areas with artifacts, necrosis or sTIL around ductal carcinoma in situ or normal tissue were excluded. Evaluation of immunohistochemistry for estrogen receptors (ER), progesterone receptors (PgR), HER2 and Ki-67 was performed following standard validated procedures. Definition of a luminal B-like tumor was based on a high Ki-67 level (defined as Ki-67 > 14%) or grade 3 or negative PgR (defined as <20%) [2]. High clinical risk was defined according to Adjuvant! criteria: T size > 3 cm; N+ with grade 1 and T size > 2 cm; N+ with grades 2–3 and any T size; grade 2 with T2N0 or higher TN stage; and grade 3 with N0 and T size > 1 cm).

### 2.3. Statistical Analysis

We followed the REMARK guidelines for the analysis and reporting of our results [25] (Appendix A). Analyses of sTIL were performed both as a continuous variable and as a dichotomic variable using two prespecified cut-offs: 10% (≤10% vs. >10%) and 0% (classification as absence of sTIL or presence of any amount of sTIL). The comparisons between groups were made with the Mann–Whitney U test or Kruskal–Wallis test for continuous variables, and with the Chi squared test for categorical variables. The association of sTIL with other variables was tested with Spearman correlation coefficients. Association of sTIL and other variables with pathologic complete response (pCR) in patients treated with neoadjuvant chemotherapy (NCT) was evaluated with logistic regression models. The goodness of fit for each model was evaluated with the Akaike Information Criterion (AIC). Accuracy was assessed with the area under the curve from the receiver operating curve (AUC ROC) using a probability of 0.5 for pCR as cut-off. The variables considered for inclusion in multivariable models were age, type of detection, tumor size, axillary node involvement (both as dichotomic and as the number of positive nodes), progesterone receptor (PgR) expression (dichotomic), Ki67 (as a continuous variable), grade (grade 3 vs. grades 1–2) and type of chemotherapy (classified as second- or third-generation chemotherapy). The selection of variables for the model was based on clinical relevance and statistical significance in the univariable analysis.

The main outcomes were relapse-free interval (RFI), defined as the interval between the date of the first treatment (either surgery or first cycle of neoadjuvant chemotherapy) and the date of distant or locoregional invasive relapse or death by BC, and breast-cancer-specific overall survival (OS), calculated from the date of the first treatment, according to STEEP criteria [26,27]. Median follow-up was calculated with the inverse Kaplan–Meier method. Assuming a maximum censoring rate under 90%, a two-sided alpha error of 0.05 and 80% power, a sample size of 343 patients was required to detect a hazard ratio (HR) of 3.0 for RFI between two groups (1:3) defined by dichotomic sTIL. Kaplan–Meier curves were generated for each group of patients. The prognostic value of sTIL for RFI and OS was analyzed by Cox regression models. The proportionality of hazard assumption was tested with Schoenfeld’s z-test. Comparison of goodness of fit between models was made with the AIC, while discrimination was compared with the C-index (previously corrected for optimism with bootstrap). The likelihood ratio test (LRT) was used for comparison of the prognostic performance of nested predictive and prognostic models.

A *p* value of 0.05 was considered statistically significant. *p* values were adjusted for multiple comparisons with the FDR Benjamini–Hochberg test. All analyses were performed with R version 4.2.3 and RStudio (version 2023.03.0).

## 3. Results

### 3.1. Patient Characteristics and sTIL Distribution According to Clinical and Pathological Variables

A total of 345 patients with available sTIL assessment were included. Patient characteristics are shown in Table 1 and Supplementary Table S1. The median age was 52 years, and approximately half of the patients were premenopausal. Most tumors were clinically detected, with 79.4% of tumors larger than 2 cm and 52.4% with axillary lymph node involvement. According to immunohistochemical classification, the majority of patients (82.9%) corresponded to luminal subtype B and 79.7% of patients were considered to be at high clinical risk, with a substantial number of patients with locally advanced disease. 

The majority of women (83.8%) received treatment with chemotherapy, either neoadjuvant (54.2%) or adjuvant (29.6%), mainly with third-generation schedules (68.7% sequential or concurrent anthracyclines and taxanes). Total mastectomy was performed in 203 (58.8%) patients and axillary lymphadenectomy in 201 (60.4%). With a median follow-up of 72 months, breast-cancer-specific OS was 94.4% (95%CI: 0.92, 0.97) and RFI was 89.5% (95%CI: 0.86, 0.93) at 5 years. 

**Figure 1:**
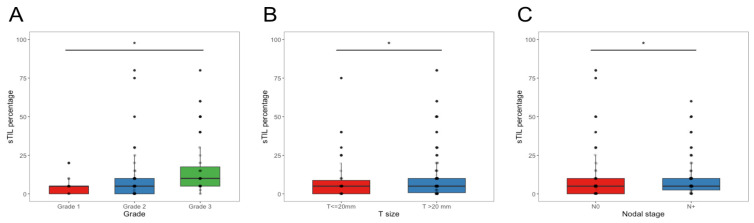
Stromal TIL proportion comparison by grade (**A**), tumor size (**B**) and axillary node involvement (**C**). The central line in each boxplot corresponds to the median value of sTIL. Black dots correspond to outliers. Error bars represent ± 1.5 IQR. * *p* < 0.05.

The median baseline sTIL infiltration was 5% (interquartile range (IQR), 0–10), and 29.0% of cases showed complete absence of sTIL (Table 1), consistent with an overall low lymphocytic infiltration (Supplementary Figure S2). Among those cases with any presence of sTIL, the majority (75.9%) presented values from 1 to 10%, and only 24.1% of tumors had sTIL greater than 10%. The proportion of sTIL (Table 1) was significantly higher in tumors of grade 3 (*p* < 0.001), larger than 2 cm (*p =* 0.004), with axillary lymph node involvement (*p* < 0.001) or of high clinical risk (*p =* 0.006) (Figure 1). Infiltration by sTIL was also associated with premenopausal status (*p =* 0.005) and younger age at diagnosis (*p <* 0.001). 

**Figure 2:**
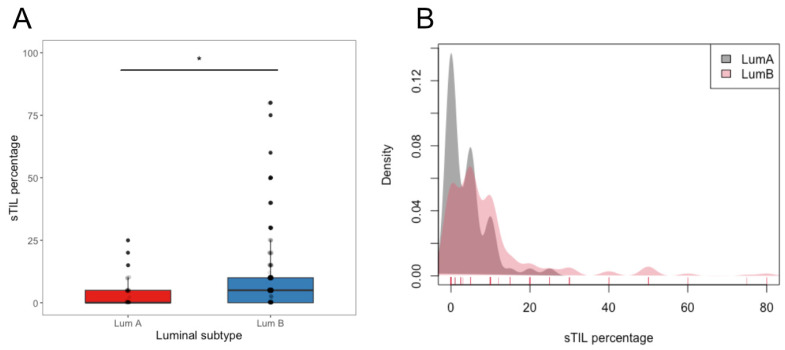
Proportion comparison (**A**) and stromal TIL distribution (**B**) according to luminal subtype. The central line in each boxplot corresponds to the median value of sTIL. * *p* < 0.005 (Kruskal–Wallis test).

The distribution of sTIL showed differences between luminal B and luminal A tumors (Figure 2), with a lower proportion of cases without lymphocytic infiltration and higher percentages of sTIL in luminal B tumors (luminal A, median: 1 (IQR 0–5); luminal B, median: 5 (IQR 1–10); *p <* 0.001). The analysis of sTIL as a dichotomic variable (sTIL = 0% or >0%) showed similar results (Table 1).

**Figure 3:**
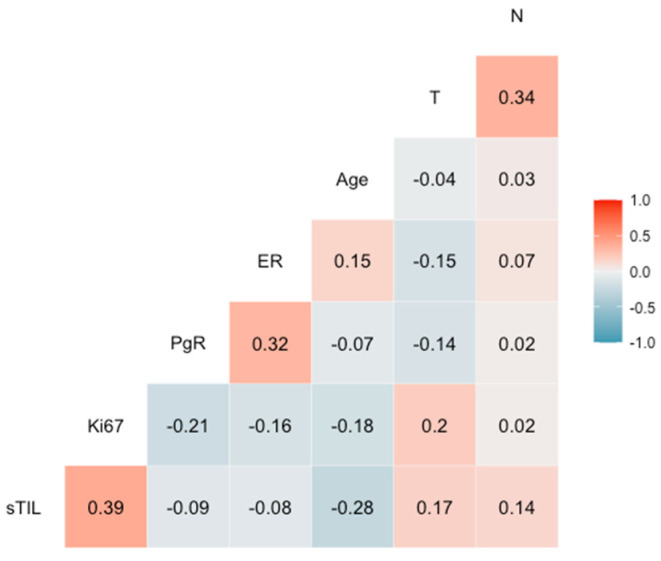
Correlogram showing the association of sTIL with other variables. Spearman correlation coefficients are shown for each pair of variables. T: tumor size (cm). N: number of positive nodes.

The percentage of sTIL was significantly correlated with a higher percentage of Ki67 (Rho = 0.391, *p <* 0.001) and with younger age (Rho = −0.28, *p <* 0.001), while it showed a weak correlation with tumor size (Rho = 0.17; *p =* 0.002) and with the number of positive nodes (Rho = 0.14, *p =* 0.012) (Figure 3).

### 3.2. Predictive Value of sTIL for pCR after Neoadjuvant Chemotherapy 

In the cohort of patients (*n* = 187) treated with NCT, the pCR rate was 8.6%. The percentage of sTIL was associated with pCR in the whole cohort of patients treated with NCT (OR = 1.055, 95%CI 1.024–1.089; *p* < 0.001). The association of other clinic-pathologic variables with pCR was only statistically significant for grade 3 and for Ki67, which showed the best predictive performance (Supplementary Table S2). The addition of sTIL to Ki67 significantly improved the performance of the predictive model for pCR (Table 2) (LRT, *p =* 0.006), with higher AUC and lower AIC values. A third model including grade 3 was not significantly better than the previous model (LRT, *p =* 0.273).

### 3.3. Association of sTIL with RFI and OS in Luminal B Tumors

**Figure 4:**
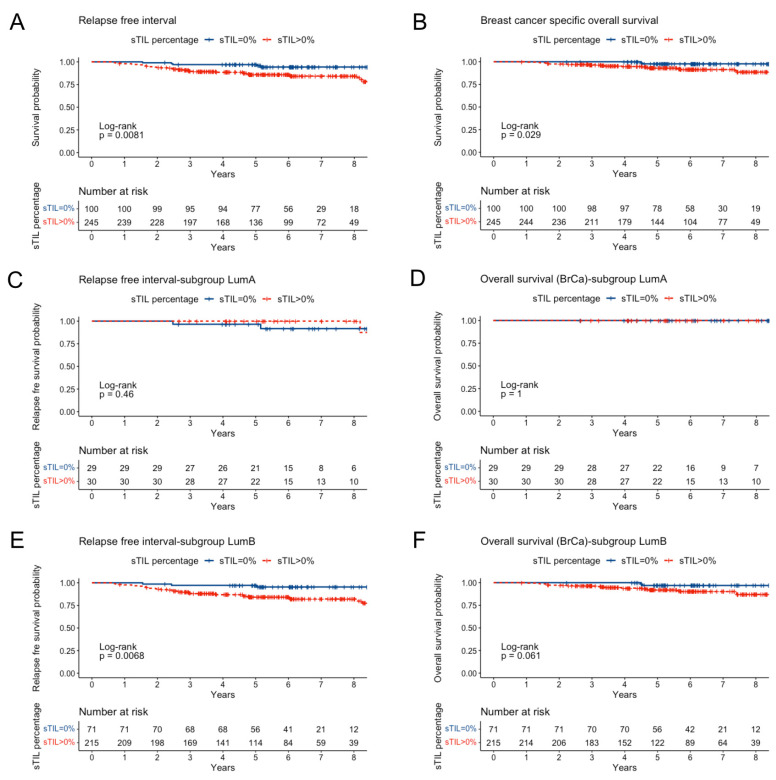
Kaplan–Meier survival curves for RFI (**A**) and breast-cancer-specific OS (**B**) for the whole group and for luminal A (**C**,**D**) and luminal B (**E**,**F**) subgroups according to sTIL (0% vs. >0%). *p* values correspond to log-rank test.

Survival analysis according to sTIL with a cut-off of 0% showed significantly better RFI (log-rank test, *p =* 0.008) and OS (*p* = 0.029) in the group of patients without sTIL presence (Figure 4A,B). Multivariate models including sTIL confirmed these results for both RFI and OS (Supplementary Tables S3 and S4). These differences were mainly driven by relapse and survival events in the luminal B subgroup (*n* = 286; 38 events, including 21 deaths by breast cancer), while only three relapses and no breast-cancer-related deaths occurred in the luminal A group (*n* = 59) (Figure 4C–F). In luminal B BC, RFI was significantly better for patients with no sTIL infiltration (HR, 4.43; 95%CI, 1.36–14.42; *p =* 0.013), while the numerical differences in OS (96.9% vs. 92% at 5 years) did not reach statistical significance (HR, 1.30; 95%CI, 0.85–15.77; *p =* 0.08). 

To specifically determine the contribution of sTIL to the prognostic performance of a multivariable Cox regression model for RFI in luminal B tumors, we built a nested model adding sTIL to a baseline model including tumor size, number of positive nodes, PgR status and Ki67. After inclusion of sTIL, the model significantly improved its performance (LRT, *p =* 0.004) (Table 3). Multivariable models for breast-cancer-specific OS did not improve their prognostic performance with the addition of sTIL (Supplementary Table S5). Differences were found neither for sTIL as a continuous variable nor for sTIL with a cut-off of 10%.

## 4. Discussion

The biological and clinical value of lymphocytic infiltration of tumor stroma in luminal breast cancer is controversial [5,10,12,13,14]. In this study, we evaluated the significance of sTIL, determined according to internationally validated criteria, in a prospective cohort of luminal BC mostly treated with neoadjuvant or adjuvant chemotherapy. In patients treated with NCT, the presence of sTIL was shown to be an independent predictive factor for pCR. However, in patients with luminal breast cancer, especially in luminal B tumors, the presence of sTIL was associated with a worse prognosis in terms of RFI and with a non-significant trend to worse OS.

Our findings reveal that there is a low percentage of sTIL in luminal cancer, with approximately one third of tumors showing no lymphocytic infiltration. This is in agreement with previous studies that have shown the immunologically “cold” microenvironment of this subtype, particularly in luminal A tumors [5]. The presence of sTIL was also associated with features of increased biological aggressiveness, such as grade 3, increased proliferation, increased nodal involvement and larger tumor size. The significantly higher sTIL percentage in premenopausal and younger women might be linked to these tumor features, which are more frequent in this population. Additionally, lower ER and PgR expression in younger patients might also justify the higher lymphocyte infiltration. These associations are also consistent with the characteristics of the luminal B subtype, which is known to be associated with higher immune activation [19,20,28]. The reasons for this higher lymphocyte infiltration are still unclear; however, a higher clonal diversity and mutational burden, reflecting a higher neoantigen expression in luminal B tumors [20], together with lower ER expression and higher proliferation, are potential explanations. Higher expression of immune checkpoint components, such as *IDO1*, in luminal B BC has also been associated with higher proliferation, lower ER expression and higher lymphocytic infiltration [28]. Therefore, substantial heterogeneity exists in the immune response of luminal BC. 

A noteworthy finding of this study is that the prognostic impact of sTIL remained independent of other clinicopathologic factors. It was possible that this prognostic effect could have been a result of sTIL being associated with other high-risk biological variables, especially those linked to proliferation. However, we specifically analyzed its impact in patients with tumors classified as luminal B according to the surrogate IHC classification. The inclusion of sTIL in a prognostic model, along with tumor size, lymph node involvement, proliferation and PgR status, resulted in improved performance, which supports the independent value of sTIL as a prognostic biomarker in luminal B and high-risk tumors. In fact, the multivariate model for RFI in luminal B tumors suggests that Ki67, although associated with sTIL presence, is a less important predictor of recurrence than PgR and sTIL. The luminal B group is particularly relevant for improving prognostic stratification and decision-making on adjuvant approaches, such as the addition of iCDK4/6 or new immunotherapy-based strategies. Moreover, the potential use of sTIL as a biomarker of endocrine resistance linked to immune activation [28], with some data indicating that sTIL could be a marker of these biological features [29], supports the possibility of its use in the context of treatment in addition to adjuvant endocrine therapy.

The dual effect of sTIL in luminal B BC is similar to that of other variables such as proliferation or high grade, which are associated with both greater chemotherapy benefit and worse prognosis. These results differ from those reported by Criscitiello et al., who reported a lower distant disease-free survival among patients with higher lymphocytic infiltration treated with chemotherapy [18], but align with those of a larger meta-analysis [14]. Despite the overall disagreement in the prognostic results, the work by Criscitiello et al. raises relevant questions concerning the impact of chemotherapy on the prognostic stratification provided by sTIL in luminal BC. According to their data, no prognostic differences by sTIL should be anticipated in the group of patients that did not receive chemotherapy, while a better prognosis for tumors with high sTIL should be observed, particularly in the subgroup with high Ki67 scores [18]. However, treatment with chemotherapy is associated, similar to higher sTIL scores, with high clinical risk, luminal B characteristics and high proliferation, thereby limiting the ability to draw firm conclusions about the differential prognostic effects between patients who received chemotherapy and those who did not. In our series, this comparison is further complicated by the low sample size of the group of patients without chemotherapy (*n* = 56). Nevertheless, we did not observe a favorable impact of high sTIL in a predominantly chemotherapy-treated population, making such an association unlikely. Another difference between our study and that by Criscitiello et al. is the substantial number of patients who received neoadjuvant chemotherapy, a therapeutic setting in which a poorer prognosis for high sTIL infiltration is supported by a recent meta-analysis [13]. The reasons for this association are unclear, as changes in the tumor microenvironment or gene expression pattern induced by chemotherapy should theoretically lead to lower proliferation and less aggressive behavior, especially in the more responsive luminal B subgroup. 

Further study of specific lymphocyte subpopulations, such as FOXP3+, macrophages and T-reg lymphocytes, among others, could provide more insights into the clinical impact of the immune response in this tumor subtype. Additionally, gene expression signatures, such as B-cell-related signatures, have shown their value as immune biomarkers, outperforming the predictive and prognostic value of sTIL in other subtypes of BC [30]. In luminal B BC, prior works have shown that genomic signatures related to tumor inflammation or BRCA-related DNA repair deficiency may also predict endocrine resistance and immune evasion, even in the presence of high sTIL [31]. While accessible, sTIL is a biologically limited marker as it can reflect different settings of immune activation or immunosuppression in luminal B tumors [28]. In fact, the greater presence of sTIL in luminal B tumors might be indicative of a higher degree of immune tolerance [20,28], which could justify the poorer prognosis observed in these patients. Therefore, sTIL should probably be complemented by other immune biomarkers to comprehensively characterize the immune tumor environment and to obtain a more accurate prognostic stratification. 

Our work has several limitations. First, the limited sample size and short follow-up, with a low number of pCR and survival events, may reduce the statistical power for the evaluation of predictive and overall survival models. Nevertheless, the fact that the majority of patients of the cohort had luminal B and high-risk tumors, mostly treated with chemotherapy, makes it more relevant from a clinical point of view and places it in the context of current development of new adjuvant or neoadjuvant treatment strategies. Second, the observational nature of the study precludes the assessment of the contribution of treatment to prognosis and of potential interactions between chemotherapy and sTIL. Third, the use of sTIL as a dichotomous variable in the survival analysis is a potential limitation. Although pre-specified cut-off points were used, the determination of sTIL is not a continuous variable because infiltration increments are usually 5% or 10%. The fact that the only cut-off point that has shown prognostic relevance has been the absence of lymphocytic infiltration versus the presence of any percentage of sTIL might suggest that the most relevant clinical factor is precisely the detection of any degree of antitumor immune response. Finally, we only evaluated pre-treatment sTIL in our work, a potential limitation since prior studies have shown the prognostic value of post-chemotherapy sTIL, especially in triple-negative BC [32]. The potential value of post-treatment sTIL has been studied less in luminal tumors, but might provide further prognostic information and improve our understanding of the interaction of chemotherapy with immune cells in the luminal BC stroma.

## 5. Conclusions

Our results show that lymphocytic infiltration in luminal breast cancer may have a different biological significance in comparison with other subtypes. While sTIL is associated with characteristics of greater biological aggressiveness and a higher rate of pathological complete response to neoadjuvant chemotherapy, it is also linked with a higher risk of relapse and breast-cancer-related death. The inclusion of lymphocyte infiltration (sTIL) improves the predictive and prognostic performance of models based on classical clinicopathological variables, even within the specific group of luminal B breast cancer. These results support the notion that the immune response plays an important role in luminal BC and suggest that sTIL might be a useful biomarker in those patients with high-risk luminal B tumors to improve prognostic stratification and therapeutic decision-making.

## Figures and Tables

**Table 1:** Baseline characteristics of population analyzed for sTIL (*n* = 345).

	Total	sTIL (%)		sTIL = 0%	sTIL > 0%	
Characteristic	*n* (%)	Median (Q1, Q3)	*p*-Value	*n* (%)	*n* (%)	*p*-Value *
Total	345 (100%)	5 (0, 10)	--	100 (29.0%)	245 (71.0%)	--
Age			--			<0.001
Median (Q1, Q3)	52 (44, 63)			57 (47, 68)	50 (42, 60)	
Menopause status			<0.001			0.005
Postmenopausal	178 (51.9%)	5 (0, 10)		64 (64.0%)	114 (46.9%)	
Premenopausal	165 (48.1%)	5 (5, 10)		36 (36.0%)	129 (53.1%)	
Histology			0.006			0.086
IDC	315 (91.3%)	5 (0, 10)		86 (86.0%)	229 (93.5%)	
ILC	27 (7.8%)	2.5 (0, 5)		13 (13.0%)	14 (5.7%)	
Other	3 (0.9%)	5 (2.5, 7.5)		1 (1.0%)	2 (0.8%)	
Subtype ^1^			<0.001			<0.001
Luminal A	59 (17.1%)	1 (0, 5)		29 (29.0%)	30 (12.2%)	
Luminal B	286 (82.9%)	5 (1, 10)		71 (71.0%)	215 (87.8%)	
PgR			0.055			0.849
Negative	44 (12.8%)	10 (0, 15)		12 (12.0%)	32 (13.1%)	
Positive	300 (87.2%)	5 (0, 10)		88 (88.0%)	212 (86.9%)	
Ki67			--			<0.001
Median (Q1, Q3)	30.0 (16.1, 41.7)			20.0 (10.0, 30.0)	35.0 (20.0, 50.0)	
Grade			<0.001			<0.001
1	52 (15.1%)	5 (0, 5)		24 (24.0%)	28 (11.4%)	
2	190 (55.1%)	5 (0, 10)		63 (63.0%)	127 (51.8%)	
3	103 (29.9%)	10 (5, 18)		13 (13.0%)	90 (36.7%)	
T stage			0.003			0.004
T1	90 (26.3%)	5 (0, 8.8)		37 (37.0%)	53 (21.9%)	
T2-4	252 (73.7%)	5 (1, 10)		63 (63.0%)	189 (78.1%)	
T size (cm)			--			<0.001
Mean (SD)	34.9 (21.6)			29.7 (20.5)	37.1 (21.7)	
Nodal stage			0.001			<0.001
N0	157 (47.6%)	5 (0, 10)		62 (62.6%)	95 (41.1%)	
N positive	173 (52.4%)	5 (2.5, 10)		37 (37.4%)	136 (58.9%)	
Positive nodes number			--			<0.001
Mean (SD)	2.1 (3.7)			1.44 (3.4)	2.4 (3.8)	
Chemotherapy ^2^			<0.001			<0.001
No chemotherapy	56 (16.2%)	0 (0, 5)		32 (32.0%)	24 (9.8%)	
2nd generation	52 (15.1%)	5 (0, 10)		16 (16.0%)	36 (14.7%)	
3rd generation	237 (68.7%)	5 (2.5, 10)		52 (52.0%)	185 (75.5%)	
Clinical risk ^3^			<0.001			
Low	70 (20.3%)	5 (0, 5)		30 (30.0%)	40 (16.3%)	0.006
High	275 (79.7%)	5 (0, 10)		70 (70.0%)	205 (83.7%)	

* Differences assessed with the Kruskal–Wallis test (for age, Ki67, T size and node number) and with the Chi squared test for the rest of variables; *p* values were adjusted for multiple comparisons (Benjamini–Hochberg method). ^1^ Luminal B subtype defined as grade 3 and/or PgR < 20% and/or KKi67 > 14%. ^2^ Second-generation chemotherapy include anthracyclines or taxanes; third generation defined as combinations (sequential or concomitant) of anthracyclines and taxanes. ^3^ High clinical risk defined according to Adjuvant! criteria (T > 3 cm; N+ with grade 1 and T > 2 cm; N+ with grades 2–3 and any T; grade 2 with T2N0 or higher TN stage; grade 3 with N0 and T > 1 cm). IDC: invasive ductal carcinoma. ILC: invasive lobular carcinoma.

**Table 2:** Association of sTIL with pCR after neoadjuvant chemotherapy.

Models	OR (95% CI)	*p*-Value	AIC	AUC ROC	LRT *p*-Value
Model 1 = Ki67					
Ki67 (continuous)	1.06 (1.03, 1.09)	<0.001	87.5	0.822	1 (reference)
Model 2 = Ki67 + sTIL					
Ki67	1.05 (1.02, 1.09)	<0.001	75.9	0.876	Model 2 vs. model 1, *p* = 0.006
sTIL (continuous)	1.05 (1.01, 1.08)	0.005			
Model 3 = Ki67 + sTIL + grade					
Ki67	1.04 (1.01, 1.08)	<0.011	76.7	0.872	Model 3 vs. model 2, *p* = 0.273
sTIL	1.04 (1.01, 1.08)	0.021			
Grade 3	2.64 (0.48, 20.50)	0.300			

AIC: Akaike Information Criteria. AUC ROC: area under the curve of receiver operating curve. LRT: likelihood ratio test.

**Table 3:** Inclusion of sTIL in Cox prognostic models for RFI of luminal B breast cancer.

Models	HR (95% CI)	*p*-Value	AIC	C-Index	LRT *p*-Value
Model 1 = T size + N+ (nr) + Ki67 + PgR					
T size (cm)	1.02 (1.01, 1.03)	0.002	323.5	0.76	1 (reference)
Number of positive nodes	1.09 (1.04–1.16)	0.001			
Ki67 (continuous)	1.01 (0.99, 1.03)	0.060			
PgR (positive)	0.47 (0.21, 1.03)	0.059			
Model 2 = T size + N+ (nr) + Ki67 + PgR + sTIL					
T size (cm)	1.02 (1.01, 1.03)	<0.001	317.4	0.75	Model 2 vs. model 1, *p =* 0.004
Number of positive nodes	1.09 (1.04–1.16)	0.001			
Ki67 (continuous)	1.01 (0.99, 1.03)	0.336			
PgR (positive)	0.36 (0.16, 0.82)	0.015			
sTIL (>0%)	4.85 (1.33, 17.65)	0.016			

AIC: Akaike Information Criteria. AUC ROC: area under the curve of receiver operating curve. C-index: Harrel’s concordance index. LRT: likelihood ratio test.

## Data Availability

The data that support the findings of this study are available from the corresponding author upon reasonable request.

## Supplementary Materials

The following are available online at https://www.mdpi.com/article/10.3390/cancers15102846/s1, Figure S1: Cohort diagram of the study, Figure S2: Histogram of frequencies of sTIL score (percentage determined by standard methods [23,24]) in luminal breast cancer. The red line represents the normal curve, reflecting a non-normal distribution. Table S1: Additional patient characteristics and sTIL distribution, Table S2: Univariable logistic regression models for prediction of pCR after neoadjuvant chemotherapy in luminal breast cancer (*n* = 187), Table S3: Multivariable Cox regression models for RFI in luminal breast cancer, Table S4: Multivariable Cox regression models for OS in luminal breast cancer, Table S5: Multivariable Cox regression models for OS in luminal B breast cancer.

## Appendix A. REMARK Criteria Checklist

**Table:**

Item to Be Reported		Page No.
INTRODUCTION		
1	State the marker examined, the study objectives, and any pre-specified hypotheses.	2–3
MATERIALS AND METHODS		
Patients		
2	Describe the characteristics (e.g., disease stage or co-morbidities) of the study patients, including their source and inclusion and exclusion criteria.	3
3	Describe treatments received and how chosen (e.g., randomized or rule-based).	3
Specimen characteristics		
4	Describe type of biological material used (including control samples) and methods of preservation and storage.	3
Assay methods		
5	Specify the assay method used and provide (or reference) a detailed protocol, including specific reagents or kits used, quality control procedures, reproducibility assessments, quantitation methods, and scoring and reporting protocols. Specify whether and how assays were performed blinded to the study endpoint.	3
Study design		
6	State the method of case selection, including whether prospective or retrospective and whether stratification or matching (e.g., by stage of disease or age) was used. Specify the time period from which cases were taken, the end of the follow-up period, and the median follow-up time.	3
7	Precisely define all clinical endpoints examined.	3
8	List all candidate variables initially examined or considered for inclusion in models.	3
9	Give rationale for sample size; if the study was designed to detect a specified effect size, give the target power and effect size.	
Statistical analysis methods		
10	Specify all statistical methods, including details of any variable selection procedures and other model-building issues, how model assumptions were verified, and how missing data were handled.	4
11	Clarify how marker values were handled in the analyses; if relevant, describe methods used for cutpoint determination.	4
RESULTS		
Data		
12	Describe the flow of patients through the study, including the number of patients included in each stage of the analysis (a diagram may be helpful) and reasons for dropout. Specifically, both overall and for each subgroup extensively examined report the numbers of patients and the number of events.	4 Figure S1
13	Report distributions of basic demographic characteristics (at least age and sex), standard (disease-specific) prognostic variables, and tumor marker, including numbers of missing values.	4–6
Analysis and presentation		
14	Show the relation of the marker to standard prognostic variables.	6–7
15	Present univariable analyses showing the relation between the marker and outcome, with the estimated effect (e.g., hazard ratio and survival probability). Preferably provide similar analyses for all other variables being analyzed. For the effect of a tumor marker on a time-to-event outcome, a Kaplan–Meier plot is recommended.	6–7
16	For key multivariable analyses, report estimated effects (e.g., hazard ratio) with confidence intervals for the marker and, at least for the final model, all other variables in the model.	7–9
17	Among reported results, provide estimated effects with confidence intervals from an analysis in which the marker and standard prognostic variables are included, regardless of their statistical significance.	7–9
18	If done, report results of further investigations, such as checking assumptions, sensitivity analyses, and internal validation.	7–9
DISCUSSION		
19	Interpret the results in the context of the pre-specified hypotheses and other relevant studies; include a discussion of limitations of the study.	9–11
20	Discuss implications for future research and clinical value.	10–11
