# Supplementary material for: Clinical Meaning of Stromal Tumor Infiltrating Lymphocytes (sTIL) in Early Luminal B Breast Cancer
Source: Cancers (Basel). 2023 May 20;15(10):2846. doi: 10.3390/cancers15102846 (PMC10216816; doi:10.3390/cancers15102846)
Supplement: Supplementary file 1 [file cancers-15-02846-s001.zip › cancers-2374246-supplementary.pdf]

SUPPLEMENTARY MATERIALS

SUPPLEMENTARY FIGURES

Figure S1. Cohort diagram of the study.

Figure S2. Histogram of frequencies of sTIL score in luminal breast cancer**.**

SUPPLEMENTARY TABLES

Table S1. Additional patient characteristics and sTIL distribution.

Table S2. Univariable logistic regression models for prediction of pCR after neoadjuvant chemotherapy in luminal breast cancer (N=187).

Table S3. Multivariable Cox regression models for RFI in luminal breast cancer,

Table S4. Multivariable Cox regression models for OS in luminal breast cancer.

Table S5. Multivariable Cox regression models for OS in luminal B breast cancer.

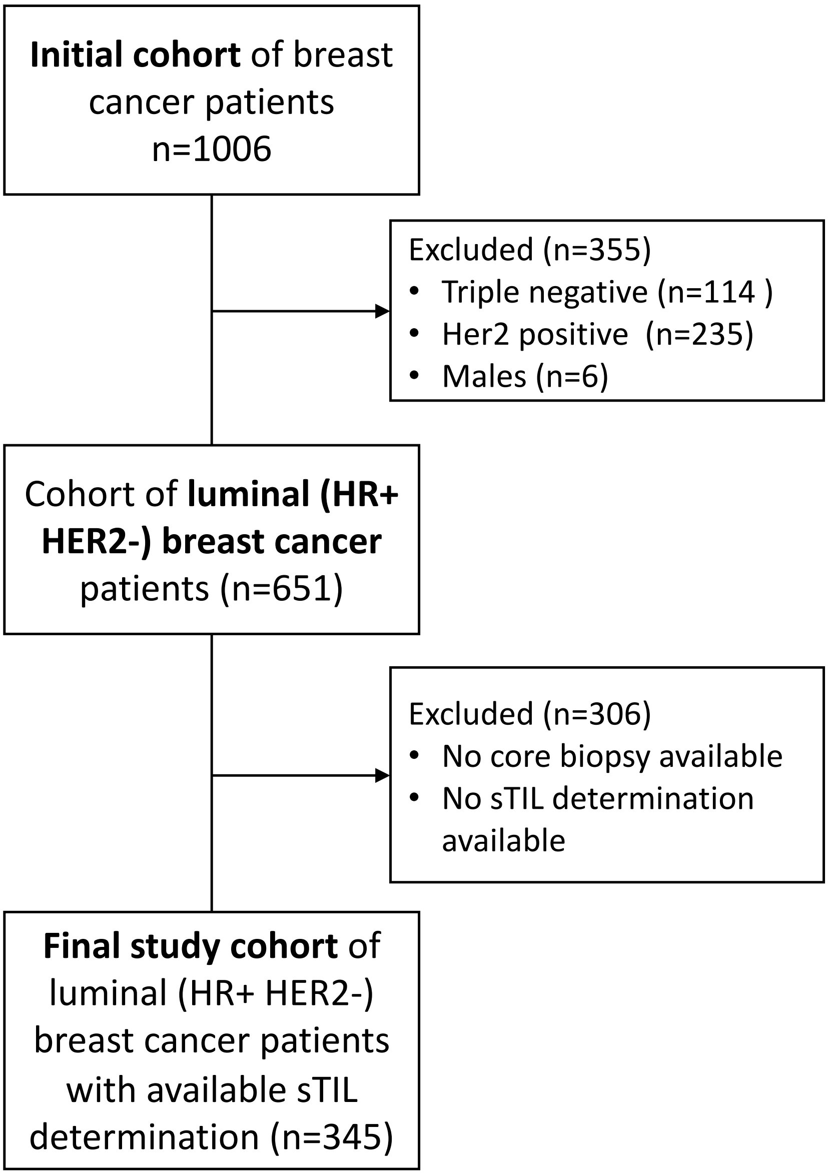

Supplementary Figure S1. Consort diagram of the study.

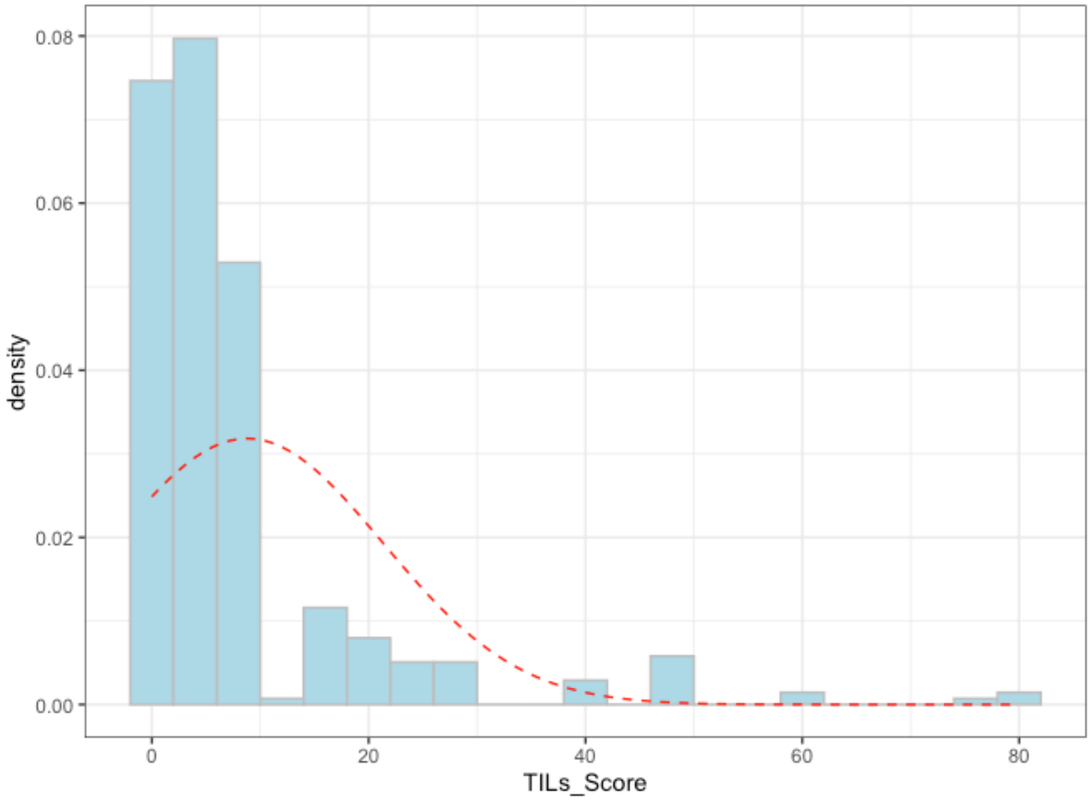

Supplementary Figure S2. Histogram of frequencies of sTIL score (percentage determined by standard methods [23, 24]) in luminal breast cancer. The red line represents the normal curve, reflecting a non-normal distribution.

Supplementary Table S1. Additional patient characteristics and sTIL distribution

Characteristic	Total N (%)	sTIL Median (Q1, Q3)	*P* value*	sTIL=0% N (%)	sTIL >0% N (%)	*P* value*
**Total**	345 (100%)	5 (0, 10)	--	100 (29.0%)	245 (71.0%)	--
**Detection**			<0.001			< 0.001
Symptoms	298 (86.4%)	5 (2.5, 10)		69 (69.0%)	229 (93.5%)	
Screening	47 (13.6%)	0 (0, 5)		31 (31.0%)	16 (6.5%)	
**Ki67**						< 0.001
<10%	57 (16.5%)	1 (0, 5)	<0.001	28 (28.0%)	29 (11.8%)	
>=10%	288 (83.5%)	5 (1, 10)		72 (72.0%)	216 (88.2%)	
**LVI**			0.880			0.997
No	161 (68.2%)	5 (0, 10)		58 (68.2%)	103 (68.2%)	
Yes	75 (31.8%)	5 (0, 10)		27 (31.8%)	48 (31.8%)	
**pN/ypN**			0.69			< 0.001
pN0	107 (31.1%)	0 (0, 5)		56 (56.0%)	51 (20.9%)	
pN1	37 (10.8%)	0 (0, 10)		19 (19.0%)	18 (7.4%)	
pN2	13 (3.8%)	5 (0, 5)		6 (6.0%)	7 (2.9%)	
pN3	1 (0.3%)	0 (0, 0)		1 (1.0%)	0 (0.0%)	
ypN0	49 (14.2%)	10 (5, 25)		5 (5.0%)	44 (18.0%)	
ypN1	71 (20.6%)	10 (5, 10)		7 (7.0%)	64 (26.2%)	
ypN2	37 (10.8%)	5 (5, 10)		4 (4.0%)	33 (13.5%)	
ypN3	15 (4.4%)	5 (5, 10)		1 (1.0%)	14 (5.7%)	
ypNx	14 (4.1%)	10 (6.2, 19)		1 (1.0%)	13 (5.3%)	
**Setting**						< 0.001
Neoadjuvant	187 (54.2%)	10 (5, 15)	<0.001	18 (18.0%)	169 (69.0%)	
Adjuvant	158 (45.8%)	0 (0, 5)		82 (82.0%)	76 (31.0%)	

*Differences assessed with the the Chi^2 test ; P values were adjusted for multiple comparisons (Benjamini-Hochberg method).

LVI: lympho-vascular invasion. pN/ypN: pathological N (node) stage.

Supplementary Table S2. Univariable logistic regression models for prediction of pCR after neoadjuvant chemotherapy in luminal breast cancer (N=187).

**Model**	**OR (95% CI)**	***P*-value***	**AIC**	**AUC ROC**
Histology (lobular)	0.00 (--)	0.318	111.6	0.526
Luminal B subtype	1.66 (0.30, 30.9)	0.613	113.0	0.518
PgR positive	0.29 (0.10, 1.01)	0.103	109.3	0.597
Ki67 (continuous)	1.06 (1.03, 1.09)	<0.001	87.5	0.822
Grade 3	4.77 (1.59, 17.6)	0.021	105	0.682
cT size (continuous, cm)	0.99 (0.96, 1.01)	0.567	111.9	0.513
cN		0.613	113.6	0.582
cN1	0.43 (0.10, 1.74)			
cN2-3	0.61 (0.18, 2.18)			

**P*-values corrected for multiple comparisons (Benjamini-Hochberg method).

PgR: progesterone receptors. cT size: clinical tumor (cT) size (cm). cN: clinical nodal stage.

Supplementary Table S3. Multivariable Cox regression models for RFI in luminal breast cancer.

Models	HR (95% CI)	*p*-value	AIC	C-index	LRT *p*-value
**Model 1 = T size + N+ (nr) + PgR**					
T size (cm)	1.02 (1.00, 1.03)	0.003	399.0	0.76	Model 1 (reference)
Number of positive nodes	1.09 (1.03, 1.15)	0.001			
PgR (positive)	0.39 (0.19, 0.84)	0.016			
**Model 2 = T size + N+ (nr) + PgR + sTIL**					
T size (cm)	1.02 (1.01, 1.03)	0.002	393.1	0.75	Model 2 vs model 1, *p*=0.005
Number of positive nodes	1.09 (1.04-1.15)	0.001			
PgR (positive)	0.33 (0.16, 0.69)	0.004			
sTIL (>0%)	3.38 (1.28, 8.99)	0.014			

PgR: progesterone receptors. cT size: clinical tumor (cT) size (cm).

Supplementary Table S4. Multivariable Cox regression models for OS in luminal breast cancer.

**Models**	**HR (95% CI)**	***p*-value**	**AIC**	**C-index**	**LRT p-value**
**Model 1 = N+ (nr) + KI67**					
Number of positive nodes	1.13 (1.05-1.21)	< 0.001	186.6	0.79	Model 1 (reference)
Ki67 (continuous)	1.04 (1.02, 1.06)	< 0.001			
**Model 2 = N+ (nr) + KI67 + sTIL**					
Number of positive nodes	1.12 (1.04-1.20)	0.002	186.8	0.80	Model 2 vs model 1, *p*=0.19
Ki67 (continuous)	1.03 (1.01, 1.06)	0.001			
sTIL (>0%)	2.45 (0.55, 10.93)	0.238			

Supplementary Table S5. Multivariable Cox regression models for OS in luminal B breast cancer.

Models	HR (95% CI)	*p*-value	AIC	C-index	LRT p-value
**Model 1 = T size + N+ (nr) + KI67 + PgR**					
T size (cm)	1.01 (0.99, 1.03)	0.199	185.1	0.77	Model 1 (reference)
Number of positive nodes	1.12 (1.04-1.21)	0.002			
Ki67 (continuous)	1.03 (1.01, 1.05)	0.007			
PgR (positive)	0.46 (0.16, 1.34)	0.15			
**Model 2 = T size + N+ (nr) + KI67 + PgR + sTIL**					
T size (cm)	1.01 (0.99, 1.03)	0.179	185.3	0.79	Model 2 vs model 1, p=0.185
Number of positive nodes	1.12 (1.04-1.20)	0.003			
Ki67 (continuous)	1.03 (1.00, 1.05)	0.022			
PgR (positive)	0.43 (0.15, 1.28)	0.131			
sTIL (> 0%)	2.49 (0.55, 11.19)	0.233			

PgR: progesterone receptors. cT size: clinical tumor (cT) size (cm).
